# Biofeedback with heart sounds following a traumatic event

**DOI:** 10.3389/fpsyg.2026.1818000

**Published:** 2026-09-08

**Authors:** Fatma Özcan, Ahmet Alkan, Nandu Goswami, Abdullah Yıldırım, Mahmut Nedim Ekersular, Büşra Oğuzhan

**Affiliations:** 1 Biophysics Department in Faculty of Medicine, Kahramanmaras Sutcu Imam University, Kahramanmaraş, Türkiye; 2 Kahramanmaras Sutçu Imam University, Department of Electrical and Electronics Engineering, Kahramanmaras, Türkiye; 3 Lodz University of Technology, Institute of Electronics, Lodz, Poland; 4 Gravitational Physiology and Medicine Research Unit, Division of Physiology and Pathophysiology, Otto Loewi Research Center of Vascular Biology, Immunity and Inflammation, Medical University of Graz, Graz, Austria; 5 Center for Spaceflight and Aviation Medicine, Mohammed Bin Rashid University of Medicine and Health Sciences, Dubai, United Arab Emirates; 6 Psychiatry Department in Faculty of Medicine, Kahramanmaras Sutcu Imam University, Kahramanmaraş, Türkiye

**Keywords:** allostatic, bio-feedback, fNIRS, phonocardiogram, trauma

## Abstract

**Background:**

Some countries have experienced and may continue to experience severe devastation due to wars, epidemics, earthquakes, and other natural catastrophes. When individuals are exposed to specific psychological or physical traumas, they can develop stress-related disorders, which can lead to irreversible changes in the autonomic nervous system and the development of cardiovascular disorders. When subjected to a new stress test, traumatized participants or those previously exposed to trauma may show more pronounced behavioral changes, intolerance, and greater fatigue responses than before trauma exposure. To reduce the recurrence of trauma-associated responses, particularly among young people, it is necessary to implement preventive measures. Alternative approaches, including counseling, medication, phytotherapy, and other interventions, need to be developed. This study aims to examine how individuals cope with trauma-related situations using a biofeedback method that could be developed to manage stress-related responses. The emotional and psychological states of earthquake victims will be assessed using a protocol based on biomedical (neurocardiac) signals and the sound of the participants’ hearts. This sound will be delivered at a fixed frequency or in real time with allostatic auditory stimulation. This approach aims to induce a resonance that stabilizes the autonomic nervous system by providing biofeedback to participants.

**Methods:**

To examine different states, biological signals will be measured and recorded, including electrocardiography (ECG), phonocardiography (PCG), electrodermal activity (EDA), respiratory rhythm, and functional near-infrared spectroscopy (fNIRS). Statistical methods and deep learning models will be applied to process the collected data optimally and evaluate the results.

**Impact:**

This study aims to assess the medium- to long-term risk of trauma-induced stress among young individuals. The use of non-invasive sound therapy to evaluate its effects on the vagal nerve through cardiovascular responses and neural activity may provide biofeedback for these individuals.

**Clinical trial registration:**

https://clinicaltrials.gov/study/NCT06919055. The trial has been registered on www.ClinicalTrials.gov with registration number NCT06919055 on 17 March 2025 with the title “Biofeedback with Heart Sound Following Trauma (ALLOSTAT0001).”

## 1 Background

People who have experienced a major earthquake, pandemic, or war often find themselves in an unstable situation, facing losses, disorganization, inability to remain in their homes, fear of another earthquake, pandemic, war, and air pollution. Young people who experience natural disasters and/or other traumatic events may develop stress-related disorders that are not always visible and may emerge over time (Scott and Weems, 2014). Furthermore, stressful life events, including those linked to natural disasters that cause trauma and stress, may precede neurocognitive disorders in people who experienced these events during their youth. In her study, Giannouli (2024) examines research conducted not only in older adults but also in animal models, as well as the associated neural mechanisms. Young people may not realize the seriousness of the situation in their daily lives. However, this may lead to changes in the autonomic nervous system and cardiac problems that cannot be adequately compensated for in the future. Adverse reactions can also be manifested by overactivity of the sympathetic nervous system or an inability of the parasympathetic system to stop overactivation. Therefore, studies assessing vagal tone at rest in young people are needed (Weems, 2015). There is growing evidence that treatments to reduce adverse outcomes and prepare young people to cope with stressful situations will improve emotional and physical health and wellbeing and provide wider societal benefits (Patron et al., 2021). The demographic and cultural differences across populations also have an impact in this regard. In this case, our study will aim to stabilize the autonomic nervous system in young people experiencing new stress.

The limitations of current stress treatments include side effects, addiction risk, time constraints, and lack of efficacy. Although cognitive therapy and psychopharmacological treatments are practical and complementary and alternative medical approaches are widely used, new and additional treatments are needed (Tegeler et al., 2023). There is evidence that listening to music may have a therapeutic effect in many areas, such as combating stress, anxiety, and sleep (Giannouli, 2017; Liu et al., 2016; Nakajima et al., 2016). Raglio and Vico suggested that the complexity of music should be simplified, the musical stimulus should be standardized, and the cultural dimension should be overcome and personalized (Adiasto et al., 2022; Raglio and Vico, 2017). Lee et al. converted certain frequencies ranging from 0.5 Hz to 48.5 Hz into audible sound in real time and then applied them to participants in the training group (Lee et al., 2019). On the other hand, the literature suggests that low-frequency sounds associated with electroencephalography (EEG) signals are suitable for treating insomnia (Gerdes et al., 2013; Guannan et al., 2023; Tegeler et al., 2023). Bhavsar et al. examined the applications of musical neurofeedback in the literature. This study presents the comparative effects of musical applications in EEG-based neurofeedback for diagnosis, treatment, and improvement. Neurological disorders after various traumas (in the EEG alpha, beta, and theta bands) were addressed through music-based neurorehabilitation, and changes in the emotional states of the participants were examined with positive results. The study also examined the effect of music on physiological parameters, such as heart rate and blood pressure, among others (Bhavsar et al., 2024). Neurofeedback is a promising approach that allows feedback to guide brain responses. The current systems are far from an individualized approach. A study presented an individualized system by testing gamma-band activity induced by auditory stimulation (Mockevicius et al., 2024). Gouda and Andrysek investigated the applicability of the Rhythmic Auditory Stimulation technique for rehabilitating temporal gait asymmetry. The study shows that the Rhythmic Auditory Stimulation Biofeedback system is promising for correcting abnormalities in gait symmetry and lower extremity disorders such as gait disorders (Gouda and Andrysek, 2024). It has been observed that such sounds are also suitable for post-traumatic stress (Lee et al., 2019).

Mechanical, vibroacoustic, or electrical/magnetic stimuli, applied in a pulsed manner to the body or to cells at frequencies close to the heart rate (approximately 1 to 3 Hz), have yielded positive results, highlighting the possible existence of a resonance effect (Kim et al., 2023; Zabrecky et al., 2020). Low-frequency mechanical stimulation has been shown to have significant effects on parasympathetic activity and heart rate variability, notably through the activation of the vagus nerve via low-frequency vibrations (Hauser et al., 2025) and transcutaneous electrical stimulation at frequencies as low as 1 Hz (Yokota et al., 2022), although these more recent studies report a more modest effect than at higher frequencies. Kim et al. aimed to prevent autonomic nervous system imbalance resulting from dynamic movement. They used average heart rate resonance (aHRR) as an auditory stimulus and investigated its role in improving the stability and homeostasis of the autonomic nervous system (ANS). The effect of aHRR on the ANS was analyzed using photoplethysmogram data collected from 22 participants by examining heart rate variability in the temporal and frequency domains. These results suggest that aHRR may influence the cardiovascular system by facilitating physiological changes that occur during dynamic movement (Kim et al., 2023). However, these studies did not replicate a stressful situation or examine the neurocardiac axis, nor did they allow for real-time monitoring of brain function and emotional and cognitive changes using fNIRS measurements. Our study proposes the exploratory hypothesis that auditory stimulation close to the natural heart rate (<3 Hz) could activate associated vagal afferent mechanisms; however, the effect of real-time heart sounds has not yet been directly tested as an auditory stimulus in the existing literature.

The body makes adjustments to maintain homeostatic balance through allostatic mechanisms; however, when stress exposure becomes exaggerated and prolonged, it may result in the failure or dysregulation of these systems (Suvarna et al., 2020). All of these conditions can be associated with autonomic imbalance, with an increase in the sympathetic nervous system (Nagai et al., 2022). The neurocardiac axis provides a physiological explanation linking psycho-emotional stressors to cardiac events (Fioranelli et al., 2018). In the neurocardiac axis, biofeedback can promote synchronization and resonance with an individual’s physiological rhythm. Kim et al. used mean heart rate resonance as a sound stimulus during dynamic movements in healthy subjects (Kim et al., 2023). However, this study did not examine oxygenated and deoxygenated hemoglobin (HbR) in different brain lobes using fNIRS measurements. Coulter et al. investigated the use of the heart rate variability biofeedback technique in managing high stress levels among people with autism spectrum disorder (Coulter et al., 2024). In patients with type 2 diabetes mellitus, the sympathetic and parasympathetic systems exhibit imbalanced functioning. This causes weakness in cardiac activity and glycemic control. In addition, the emotional state of patients may also be negatively affected. Wu et al. used the heart rate variability biofeedback technique in their study and proved its effect on these adverse outcomes (Wu et al., 2024). Ramakumar and Sama (2024) examined the effect of heart rate bariability biofeedback on perioperative stress, recovery, and anesthesia management in surgical patients. Heart rate variability is a physiological marker through which the positive or negative consequences of the activities of the autonomic nervous system can be observed. The resonance state is primarily associated with vagal nerve activity (Ramakumar and Sama, 2024). Some studies have reported improvements in insomnia, postnatal depression, stress, and anxiety disorders with biofeedback (Blase et al., 2021; Gerdes et al., 2013; Lee et al., 2019; Tegeler et al., 2023).

In the literature, stress generation in individuals can be achieved through the mental arithmetic (Goswami et al., 2012; Mezzacappa et al., 2001), social noise (Liu et al., 2016; Nakajima et al., 2016), and dynamic movement (Kim et al., 2023; Lampert, 2015). The recovery heart rate of subjects performing mental arithmetic tasks was lower than the baseline heart rate (Goswami et al., 2010), and the heart period variability was higher than the baseline. Cardiovascular recovery from stress is associated with increased vagal modulation despite sympathetic activation. These findings confirm the association between vagal reflex attenuation and cardiovascular disease risk (Mezzacappa et al., 2001). In this study, we will introduce new mental stress levels (Lampert, 2015) for younger individuals.

In this context, there have not been many studies on new techniques, such as deep learning techniques currently used in signal and image processing. As part of this study, statistical tools will first be used, followed by deep learning models, to process the data collected and evaluate the results as comprehensively as possible. Explainable artificial intelligence (XAI) techniques will enable this approach to be presented in a transparent manner (Ong et al., 2021). Thus, the interpretability of the selected models can contribute to the design of efficient classifiers.

Our study investigates how individuals can cope with such stress-related disorders and what kind of biofeedback can be developed to manage them. In order to facilitate the balancing and self-calibration of neuronal oscillations, this study, within the scope of the biofeedback study, will create awareness by presenting the participant’s heart sound as a warning in real time. As the heart rhythm increases, the person will perceive that he/she needs to self-regulate and calm down through biofeedback, thereby improving stress management and affecting the autonomic nervous system. In summary, allostatic regulation and autonomic balancing of neural oscillations in trauma-exposed individuals will be explored non-invasively through biofeedback.

## 2 Objectives

The ALLOSTAT0001 project aims to analyze and investigate cardiovascular risk in young people who have experienced trauma, such as that caused by an earthquake. The aim of this study is to develop biofeedback for non-invasive relaxation and autonomic stabilization of neural oscillations in young participants with post-traumatic stress. Using a framework that employs mental stress tasks and auditory stimuli derived from heart sounds, we will investigate whether biofeedback can be enhanced by supporting allostatic regulation. This may lead to synchronization and resonance with an individual’s physiological rhythm.

The study will also provide a better understanding of the physiological responses (electrodermal activity and neuronal activity) of young participants undergoing another type of mental stress. In this context, young participants’ emotional and psychological states will be observed using biomedical signals, and processing and relaxation states will be derived from these signals. Repeated measurements over a given period will provide valuable information regarding changes in cardiovascular and psychological risk markers over time.

While some studies have attempted to reduce the allostatic load by applying auditory stimuli derived from electroencephalogram signals or stimuli with a musical tone, there is currently very little information on applying auditory stimuli derived from heart rate. The use of stimuli with a frequency that has the potential to induce resonance and measurements of oxygenation in the prefrontal cerebral area using the fNIRS technique enhances the novelty of the project and underlines its strengths.

In this interdisciplinary study, we aim to use the measured heartbeat sound as an auditory stimulus and examine its effect on the stability of the autonomic nervous system in young participants exposed to mental stress. A young person entering a new mental stress environment will be given their heart sound (or a metronome synchronized with their heart rhythm while calm) or a real-time heart sound as an auditory stimulus. According to the proposed hypothesis, it is assumed that this will produce a resonance effect in the participant and promote relaxation. Typically, it is believed that the average heart rate is the individual’s intrinsic resonant frequency due to the influence of the autonomic neural system (ANS). In other words, allostatic acoustic stimulation at the same frequency as the individual’s heart rate will transmit auditory information and may activate the young participant’s brainstem.

First, statistical tools and then deep learning models will be used to process the collected data optimally and evaluate the results. Explainable artificial intelligence (XAI) techniques will support the transparent interpretation of this approach. With this protocol, we aim to use signals to show the correlation and causality between the stimulus and psychophysiological response.

The aim of this study is to contribute to the literature and scientific community by obtaining research results at an international level and by clarifying unexplored elements of a topic that has not been studied in this context previously. The collected parameters have great potential for improving the prediction of future cardiovascular and psychological events in young people, adults, and children. The type of trauma addressed will not only be the experience of an earthquake but also exposure to other natural disasters, epidemics, or wars.

The information obtained from this project is important for developing guidelines and recommendations for managing the health of young people at risk. The results of the ALLOSTAT0001 project will also contribute to the search for solutions to enable young people who have suffered significant trauma to lead healthy lives over a long term. Early self-therapy using individual efforts and natural methods may be highly effective in minimizing the need for medication or hospitalization.

## 3 Methods/design

The Project has received ethics approval from Harran University with the decision of the clinical research ethics committee. The study was registered under the number E-76244175-050.04-346993, on the 10th of June 2024. The study will be conducted at Kahramanmaras Sütçü İmam University in Türkiye. The study was subsequently registered with www.ClinicalTrials.gov under the registration number NCT06919055 in March 2025 and follows the Standard Protocol Items: Recommendations for Interventional Trials (SPIRIT) reporting guidelines (Chan et al., 2013). All items from the World Health Organization Trial Registration Data Set are summarized in Table 1 according to the SPIRIT reporting guidelines. Data collection began in December 2024 and completed by 30 June 2025.

**Table 1:** Items from the World Health Organization trial registration dataset.

Section/item	Information
Primary Registry and Trial Identifying Number	ClinicalTrials.gov, NCT06919055
Date of registration in primary registry	17 March, 2025.
Secondary identifying numbers	124S102
Source(s) of monetary or material support	The Scientific and Technological Research Council of Türkiye (TÜBİTAK)
Primary Sponsor	Özcan, F. and Goswami, N
Secondary sponsor (s)	Alkan, A., Yildirim, A.
Contact for public queries	Özcan, F., fatma.ozcan13@gmail.com
Contact for scientific queries	Özcan, F., fatma.ozcan13@gmail.com and Goswami, N nandu.goswami@medunigraz.at
Public title	Allostat0001
Scientific title	Biofeedback with Heart Sound Following Trauma
Countries of recruitment	Turkey
Health condition(s) or problem(s) studied	This study aims to examine how individuals cope with trauma-related situations using a biofeedback method, which could be developed to manage stress-related coping.
Intervention(s)	1. Control 2. Heart sound stimulation 3. Metronome with ECG rate stimulation
Key inclusion and exclusion criteria	36 earthquake survivor university students aged between 20 and 25 years will participate in the experiment. Participants will be healthy, non-obese men (*n* = 18) and women (*n* = 18). In particular, all participants should have experienced an earthquake disaster in the local region. All participants should be free of any chronic or acute infection, neurological, psychiatric, and/or cardiovascular disease. They must be non-smokers, have no alcohol or drug addiction, and not be under any medical treatment. Pregnant or breastfeeding women will not be included in the study.
Study type	Allocation: non-randomized Intervention model: parallel assignment Primary purpose: treatment
Date of first enrollment	December, 2024.
Target sample size	36
Recruitment status	Recruiting.
Primary outcome(s)	Method of measurement: Biopotentials (ECG, PCG, EDA, fNIRS, respiratory rhythm) Timepoint: pre-treatment, post-treatment, and follow-up.
Key secondary outcomes	Changes in neuroimaging measures Timepoint: pre-treatment, post-treatment, and follow-up. Changes in biopotential measures Timepoint: pre-treatment, post-treatment, and follow-up.
Ethics Review	This project received ethical approval from Harran University with the decision of the clinical research ethics committee on 03 July 2024.
Completion Date	October, 2025

The SPIRIT reporting guidelines recommend these items to be included in trial protocols to provide a brief, structured overview of a trial.

Our study is a non-randomized, parallel-group cohort study. Patients were assigned to a group in the chronological order of enrollment in the study. Therefore, consecutive sampling was used for allocation concealment. The inclusion and exclusion criteria will be applied uniformly to all patients, regardless of group, without exception. At inclusion, baseline characteristics will be compared between groups, including age, sex, and whether participants experienced the earthquake as a traumatic event. The comparability of the groups at inclusion will be verified by ensuring that there are no significant differences between groups.

In order to carry out this study, the following basic measurements—sex, age, height, weight, blood pressure, etc.—and relative measurements derived from the basic measurements will be extracted. In this context, the cardiovascular, emotional, and psychological states of earthquake survivors will be observed through biomedical signals. Electrocardiography (ECG), respiratory rhythm, and electrodermal activity will be measured to evaluate the autonomic nervous system, particularly the parasympathetic nervous system. A metronome based on the heart rate derived from the ECG and the measured phonocardiogram signal will be used for auditory stimulation. Our protocol will calculate and use the biological markers of heart rate variability (HRV), including low-frequency band (LF), high-frequency band (HF), LF/HF, standard deviation of NN intervals (SDNN), and root mean square of successive differences (RMSSD). The fNIRS device will measure changes in oxygenated and deoxygenated hemoglobin concentrations in the cortical brain structure before and during mental exertion. The sound of a heartbeat will be evaluated to determine its effects on fNIRS results in the prefrontal cortex during relaxation and stress.

Each participant will have 390 min of experimental activity, including 330 min of measurements.

### 3.1 Protocol and procedure

#### 3.1.1 Inclusion criteria

Young earthquake survivors without specific diseases who recently experienced the earthquake in our region in February 2023 will be selected. The sample sizes of 20–25 participants have been selected in similar studies (Maheu et al., 2005; Nakajima et al., 2016; Tegeler et al., 2023; Zabrecky et al., 2020). The sample size selected (*n* = 10–12 participants per group) follows the rule of thumb proposed by Julious (2005), which is widely recognized in the methodological literature as a minimum standard for pilot studies (Julious, 2005). Furthermore, our proposal will use deep learning and current artificial intelligence algorithms. Because data can be obtained from each measurement within the time-series dataset used for this purpose, a substantial amount of data can be generated through windowing and data augmentation, and transfer learning can be performed; it is assumed that a dataset from 36 participants will be sufficient. Consequently, at least 30 university students aged between 20 and 25 years who experienced and survived the earthquake will take part in the experiment. They must have experienced the earthquake as a significant and severe event. In accordance with the inclusion criteria, the participants will be healthy, non-obese men (*n* = 18) and women (*n* = 18). In particular, all participants should have experienced the earthquake disaster in the local region. All participants must be free of any chronic or acute infection, neurological, psychiatric, and/or cardiovascular disease. They must be non-smokers, have no alcohol or drug addiction, and not be under any medical treatment. Pregnant or breastfeeding women will not be included in the study.

University students who survived the earthquake in the region will be invited to volunteer for this study. The inclusion and exclusion criteria will be verified by interviewing the volunteers. Participants will then undergo a medical examination by a psychiatrist. If no problems are found, participants will be recruited for the study.

#### 3.1.2 Study design

An intervention and control group will be formed with 20 and 10 participants, respectively. The intervention and control groups will be exposed to the same mental stress.

*Control group:* 10 young healthy earthquake survivors will not receive any auditory stimulation.

1. Intervention group: 10 young healthy earthquake survivors will be subjected to closed-loop, real-time heart sound (PCG) stimulation to create biofeedback.
2. Intervention group: 10 young healthy earthquake survivors will be given an auditory stimulus of their heart sound and a metronome synchronized with their mean heart rate (MHR) recorded during a calm state.

Participants will be taken individually to the experimental room and seated comfortably in a quiet and spacious environment. Before the first session, a scientific introduction, the purpose of the study, an explanation of how it will be conducted, an explanation of the mental stress to be induced, and basic information about the measurements to be taken will be provided at the information meeting (using an introduction video). The participant should feel as comfortable as possible, and no additional sources of stress should be created. Participants will be instructed to breathe normally and avoid moving their legs.

**Figure 1:**
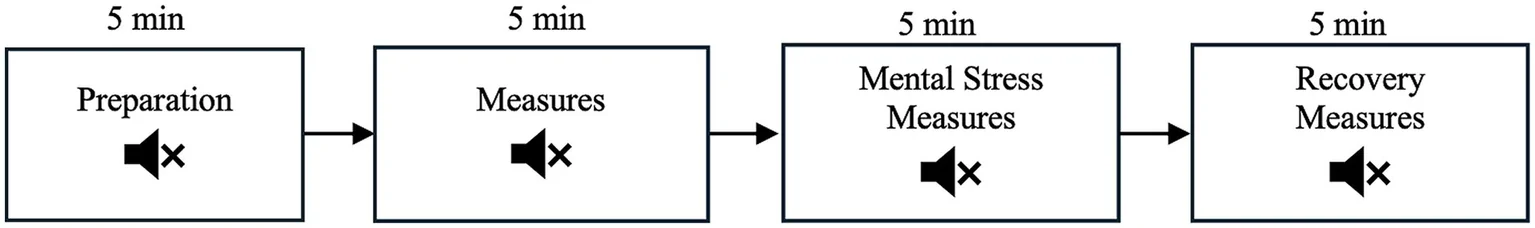
Procedure to be applied to all groups before the first session.

**Figure 2:**
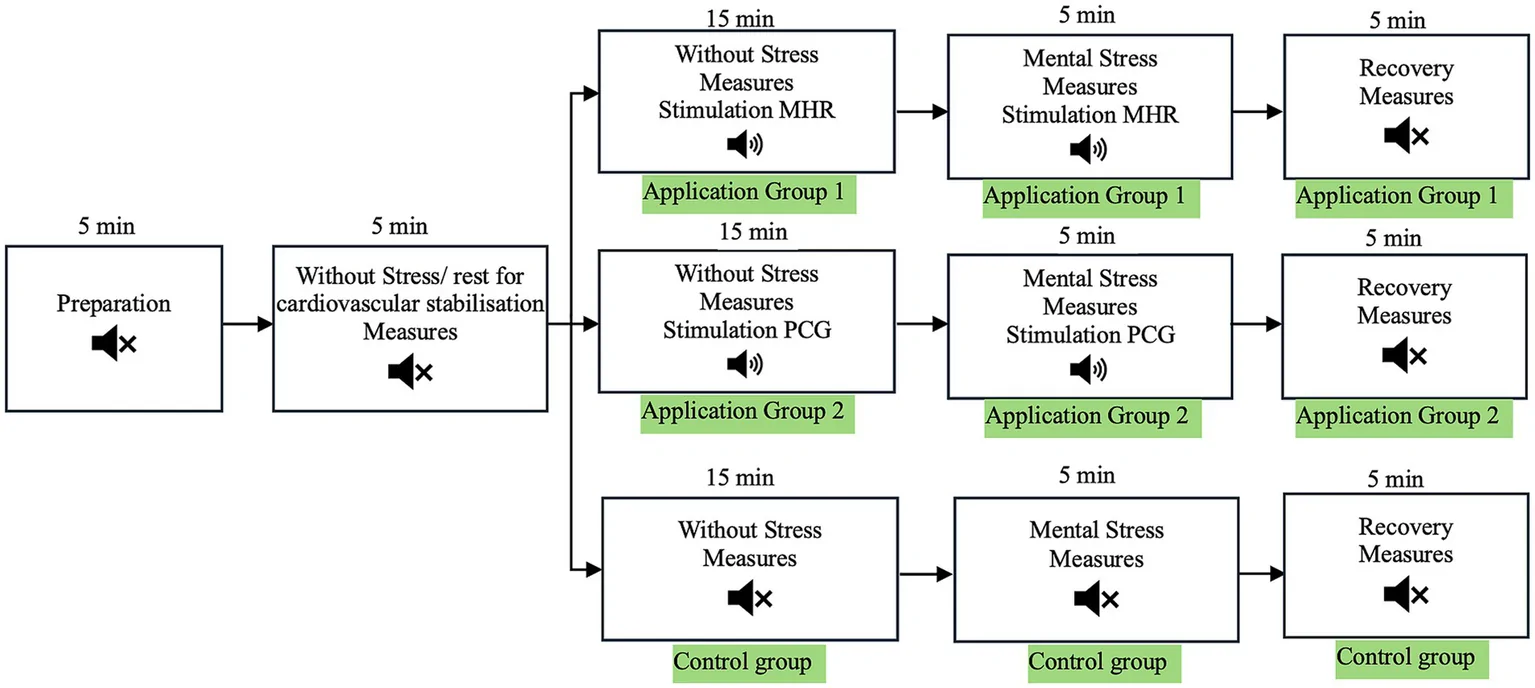
Procedure to be applied in each session (10 sessions). MHR: Stimulation at Mean Heart Rate; PCG: real-time and closed-loop heart sound stimulation.

**Figure 3:**
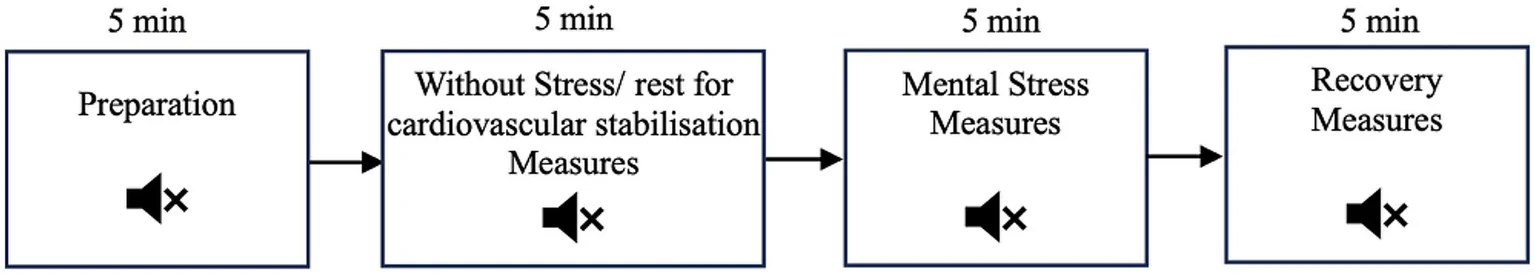
After 4 weeks, the procedure to be applied to all groups.

From the study of Gerdes et al., public treatment centers called “Cereset” were opened on the American and European continents. Similar to physiotherapy centers, these centers offer relaxation methods over 10 sessions within 4 weeks by acting on the patient’s parasympathetic system using EEG signals (Tegeler et al., 2023). For post-traumatic therapy sessions, 10 sessions of 30 min each are considered sufficient (Desbiendras, 2024). In this context, the participants will have 10 sessions over approximately 4 weeks (S1-S10). According to the literature, 10 sessions are sufficient for effective intervention and adequate observation. Participants will receive the first two sessions on consecutive days. Then, participants will have two sessions in a half-day period with a 1-h break between sessions. Participants will receive the third session within the first 5 days. Participants will complete the rest of the 10 sessions, with a maximum of 5 days between sessions. The sessions will occur between 1:00 and 5:00 p.m. ±30 min, as the participants may better remember emotionally stimulating information in the afternoon (Maheu et al., 2005). In addition, according to Figures 1–3, the transitions between stages will be direct and immediate without a break. In addition, the date of menstrual onset will be recorded to account for the menstrual periods of female participants.

In this context, earthquake victims’ emotional and psychological states will be monitored using biomedical signals, and responses will be derived from these signals. Respiratory rhythm, electrocardiography (ECG), phonocardiography (PCG) (Özcan, 2024), electrodermal activity (EDA), and functional near-infrared spectroscopy (fNIRS) measurements will be performed during the information session, at the beginning of the sessions, throughout the 10 intervention sessions, and 4 weeks after the intervention (Arsalan et al., 2023). The application after about 4 weeks is to see whether the intervention is permanent.

Stress, anxiety, and depression will be assessed using psycho-emotional stressors at the beginning and end of the intervention using the Turkish version of the Depression Anxiety Stress Scale–21 (DASÖ-21) (Yıldırım et al., 2018).

Live heart sounds or average heartbeat sounds will be used as stimuli to provide biofeedback. A metronome will also be used for the auditory stimuli, including the heart rate from the ECG and the measured phonocardiogram. The audio stimulus will be presented at an intensity of 60–65 ± 10 dB before and during mental stress.

Different mental stress tasks will be induced in all sessions and 4 weeks after the sessions. A mental arithmetic task is a mechanism that increases the mental workload by performing a series of arithmetic operations of varying degrees of difficulty. One of the effective platforms used in the literature is Millisecond (Millisecond Software, Seattle, WA, USA), and the Trier Mental Challenge Test (TMCT) available through the Inquisit application will be used in the study. Millisecond Software is a leading software provider for psychological testing. The Inquisit platform has been used in applied fields, such as clinical trials, and several peer-reviewed studies. The TMCT is a timed arithmetic test designed by Kirschbaum to induce psychological stress in participants (Kirschbaum, 1991). During the session and throughout the 10 sessions, the mental operations should be randomized in order to create stress without allowing learning to occur.

Deep learning techniques and statistical analyses will be used to examine the causality between the generated stress, given warning, and measurements. The data will be processed as a time series or converted into images in our study. Each participant will have 390 min of experimental activity, including 330 min of measurement time.

Transfer learning from an existing pretrained convolutional neural networks (CNNs) will be applied for the acquired data. If there is sufficient data to train a new neural network, the formation of a new network architecture is considered for the sustainability of the protocol.

Explainable artificial intelligence techniques will be used to analyze the data attributes used by the network.

Below are the protocol diagrams to be applied before the first session, throughout the 10 intervention sessions, and after 4 weeks (Figures 1–3).

### 3.2 Data acquisition device

Continuous monitoring of physiological signals, including respiratory rhythm, electrocardiogram (ECG), phonocardiogram, and electrodermal activity, will be performed using the BIOPAC System.

The fNIRS device from BIPAC will measure oxygenated and deoxygenated hemoglobin concentrations in the cortical brain structure during mental exertion (Santosa et al., 2014).

### 3.3 Relative measurements/parameters

The parameters obtained from the measurements and their meanings are summarized below.

- Heart rate, heart rate variability (HRV), low-frequency band (LF), high-frequency band (HF) components of the HRV signal, the LF/HF ratio, and HFnu = HF/LF + HF (normalized unit) will be calculated.
- Standard indices of vagal control: The root mean square successive differences (RMSSD) between RR intervals, standard deviation of normal-to-normal (NN) intervals representing standard deviation of normal-to-normal (NN) intervals (SDNN), and percentage of consecutive regular sinus RR intervals of more than 50 ms (pNN50; Laborde et al., 2017) will be calculated.
- Galvanic skin response, including EDA/skin conductance level (SCL) and event-related skin conductance responses (SCRs) attributable to a specific stimulus, will be used as parameters (Braithwaite et al., 2015).
- Oxygenated hemoglobin concentration (HbO), deoxygenated hemoglobin concentration (HbR), total hemoglobin concentration (HbT = HbO + HbR), and oxygen consumption (Oxy = HbO - HbR) will be used as parameters derived from fNIRS data (Ghosh, 2024).

### 3.4 Data analysis

Mood assessment will be conducted as described below using methods to analyze pre- and post-treatment questionnaires and by examining changes in parameters derived from biological signals (RMSSD, LF, HF, LF/HF, SDNN, SCL, SCR, and respiratory rate). Statistical methods will be used to analyze and evaluate the data obtained from the collected stress and anxiety questionnaires. In addition to respiratory rate, ECG, PCG, EDR signals, heart rate, heart rate variability (HRV), low-frequency band (LF) and high-frequency band (HF) indicators from the HRV signal, the LF/HF ratio, SDNN, RMSSD, and pNN50 parameters will be processed. Functional NIRS will be used to monitor brain functions in real time. These analyses will be based on changes in oxygenated and deoxygenated hemoglobin concentrations in the cerebral cortex. In particular, the prefrontal cortex region and limbic lobe (Balters et al., 2021; Dattola et al., 2023) will be monitored. Changes in the obtained signals and parameters change before, during, and after the intervention will be analyzed. Statistical techniques, deep learning, and explainable artificial intelligence (XAI) techniques will be used for this purpose. To evaluate the benefits and differences of the biofeedback study, classification analyses will be performed on the data.

#### 3.4.1 Phonocardiogram data and fNIRS data

Heart sounds to be used as stimuli and for monitoring purposes will be converted into Mel spectrograms and input into neural networks trained with sound (OpenL3, YAMNet, and VGGish), and transfer learning will be performed.

Cognitive assessment will be performed by analyzing fNIRS signals to assess activation in specific brain regions (right, center, and left). The details are provided below. During mental stress, classification analyses will be performed between the results between the control and treatment groups and between the results of the same group before and after treatment. Then, using explainable artificial intelligence (XAI) techniques, the temporal and frequency-domain characteristics associated with these changes will be evaluated.

#### 3.4.2 ECG, EDR, and fNIRS data

The data obtained using deep learning techniques will be classified. Classification studies will be performed with deep learning to emphasize to characterize differences between the control and treatment groups, as well as differences before and after treatment within groups and to show that the difference is significant. The data will be converted into spectrogram images and processed as time-series data. A suitable pretrained network (AlexNet, SqueezeNet, GoogLeNet, etc.) will be used with transfer learning, and classification will be performed. Technical information is provided in our published articles (Özcan and Alkan, 2021, 2023a, 2023b).

On the other hand, correlation analysis will be performed using deep learning method (Alessandrini et al., 2023). Then, the features used in the classification will be evaluated using explainable artificial intelligence techniques.

#### 3.4.3 Numerical parameters

Machine learning algorithms and statistical techniques will be used to analyze numerical data. The optimal technique will be selected based on the characteristics of the data. Although not a priority, some basic classical classifiers, including support vector machines [SVM], k-nearest neighbors [KNN], and ensemble methods, along with statistical analyses, will be included for comparative analysis. In such cases, feature extraction from the dataset is important. For this purpose, the RMSSD, LF, HF, LF/HF, SDNN, SCL, SCR, HbO, HbR, and Oxy parameters will be used as features.

### 3.5 Assessing the results

The analysis and evaluation of the data obtained from the collected stress and anxiety tests will be performed by the researcher physician. The supervisor and researchers will analyze and evaluate the respiratory rate and ECG, PCG, EDR, and fNIRS data. Respiratory rate, ECG, PCG, EDR, and fNIRS data will be analyzed and evaluated by the supervisor and researchers.

#### 3.5.1 Statistical methods

RMSSD, HF, and LF derived from HRV will be the primary pilot outcomes for estimating the effect size and variance, with a view to calculating the sample size for a future confirmatory trial. The primary outcomes will be assessed at the final session and compared with those obtained at the first session. The analyses will be conducted using a linear mixed-model (LMM), with group and time as fixed effects or using an analysis of covariance (ANCOVA). Other measures, such as electrodermal activity, respiratory rate, and neuroimaging, will be treated as secondary endpoints for exploratory purposes.

#### 3.5.2 Methods for deep learning

In preprocessing, the time series can be used as raw signals or converted into images depending on the type of analysis (spectrogram, melspectrogram, etc.). The Mel spectrogram is a logarithmic frequency spectrum on the Mel scale and provides a time–frequency representation of audio signals. The Mel scale approximates the scale of sound perception in the human ear (Peng et al., 2022). In particular, PCG signals will be converted into Mel spectrograms.

In deep learning, features from the input data are automatically extracted and evaluated by the network. Because audio-pretrained networks are trained on the pitch of heart sounds, it is more convenient to use these networks on fNIRS signals from the cortex when auditory stimulation is provided. For voice classification, pretrained convolutional neural networks (CNNs) are used to achieve significant success values in voice classification. These artificial networks include OpenL3, VGGish, and YAMNet (Peng et al., 2022; Tsalera et al., 2021). Our study will transfer learning from an existing pretrained CNN. If there is sufficient data to train a new neural network, the formation of a new network architecture is considered for the project’s sustainability. These networks learn to recognize features from Mel spectrogram representations of audio signals. Feature extraction in our study will be performed automatically by transfer learning (Özcan and Alkan, 2021, 2023a, 2023b). ECG and EDR time series will be handled using long short-term memory (LSTM) networks. Fully connected deep networks (dense neural networks [DNN]) will be used to evaluate the correlation between stimulus measurements and stress measurements.

Transfer learning will provide faster and more effective learning algorithms by reducing the size of the dataset. The network will be fine-tuned with transfer learning, and feature extraction will be performed quickly. The dataset will be expanded using smaller time windows of the signals. If we divide 330 min of recordings into 10-s segments, 1980 time series or representative images will be processed for each measurement. In addition, the dataset can be expanded using the data augmentation technique. Data from 36 subjects would be sufficient in this case. Of the dataset, 70% will be used for training the neural network, 15% for validation, and 15% for testing. To ensure that the training and test sets are created on an individual basis, we would need a larger number of participants. This could be the subject of future studies.

Despite their excellent performance, deep learning models produce good results without being able to explain why. These models act as a black box for users. This is one of the main limitations of deep learning in medicine. Therefore, the issue of the explainability of neural networks has emerged (Ilias and Askounis, 2022; Ong et al., 2021; Thibeau-Sutre et al., 2023). The behavior and reasons for the importance of features automatically used in artificial intelligence tools will thus be understood (Ong et al., 2021). The XAI framework will use different techniques, such as LIME (Local Interpretable Model-agnostic Explanations), Occlusion Sensitivity, and Grad-CAM. These methods, each with a specific approach to classifying an image, produce a map showing the regions of the image that are important for the process. The network will use these maps for decision-making.

#### 3.5.3 Performance metrics

The proposed methods’ accuracy, sensitivity, specificity, precision, and F1-score (Table 2) will be measured as evaluation parameters. A confusion matrix will also be extracted to provide an overview. An optimization study will be performed to determine the best hyperparameter settings. Five cross-validation will be selected, and the average values of the results will be obtained. The methods with the highest performance will be compared with the others.

**Table 2:** Calculations of performance metrics (Özcan and Alkan, 2021, 2023a, 2023b).

Accuracy	Sensitivity	Specificity	Precision	F1 score
$\frac{\mathrm{TP}+\mathrm{TN}}{\mathrm{TP}+\mathrm{TN}+\mathrm{FP}+\mathrm{FN}}$	$\frac{\mathrm{TP}}{\mathrm{TP}+\mathrm{FN}}$	$\frac{\mathrm{TN}}{\mathrm{TN}+\mathrm{FP}}$	$\frac{\mathrm{TP}}{\mathrm{TP}+\mathrm{FP}}$	$\frac{2\mathrm{TP}}{2\mathrm{TP}+\mathrm{FP}+\mathrm{FN}}$

TP: true positive; FN: false negative; FP: false positive; TN: true negative.

#### 3.5.4 Graphical user interface

Presenting a system created as project output through an interface will provide ease of use. The App Designer application will be used in MATLAB. In the user interface to be developed, the data obtained from the experimental study can be displayed in graphical and tabular forms, the desired analysis method can be selected, and the results can be displayed. Therefore, the data and results will be visualized for the study users.

## 4 Importance of study/implications of findings

The ALLOSTAT0001 project, which will use the protocol described above, will analyze and investigate cardiovascular risk in young participants who have experienced trauma, such as a natural disaster or an earthquake. Neurocardiac biofeedback to preserve allostasis may be possible by applying heart sound-based stimuli. The physiological responses (electrodermal activity and neuronal activity) of these participants subjected to another type of stress (mental) will no longer be the same as before the biofeedback treatment sessions. These young participants will, therefore, learn strategies to manage new stressors in their lives and remain calm. By following them over time, using repeated measurements of cardiovascular and psychological risk markers, we can establish guidelines and recommendations for managing the health of young people at risk.

Auditory stimuli with heart rate will reduce the allostatic load by inducing resonance. Oxygenation and deoxygenation measurements in the prefrontal region of the cerebral cortex using fNIRS will provide a better understanding of cerebral activity during new stress and its attenuation generated by our method. These data can be used in neuroscience research.

The parameters collected will make it possible to improve the prediction of future cardiovascular and psychological events, not only in young people but also in adults and even children. With significant results, this protocol can be used for people who have experienced other traumas, such as natural disasters, epidemics, or war.

## 5 Limitations

Although the ALLOSTAT0001 project provides a great deal of new information, it is associated with several limitations for which mitigation strategies can be proposed. The risks are detailed as follows.

Many young participants from the city where the earthquake occurred are studying in another city. This may limit our ability to recruit participants who survived the disaster, restricting the sample size to no more than 36 participants. We expect to recruit the number of participants needed to reach statistical significance in this study. For the study using deep learning techniques, 36 participants are expected to be sufficient when using transfer learning.

At the end of the project, missing data may occur. When collecting signals, we may notice that the signal is incomplete or has anomalies. As missing data can limit statistical comparisons between groups, we will reduce this problem by recruiting other participants. We will recruit an additional 10 participants who will provide backup data. If we cannot recruit participants aged 20–25 years because many students were absent (at the end of their school holidays) during the earthquake in 2023, we can extend the age range to 18–25 years. In addition, we will take the necessary steps in the statistical analysis plan to address the missing data.

Each participant will be invited to return 4 weeks after the final session for a follow-up evaluation. However, the start of holiday period may make it challenging to collect data after the event. We will take care to plan the sessions and the day of the follow-up evaluation, prioritizing participants who will be staying in the city during the holiday period.

This study is a preliminary investigation aimed at exploring whether real time, closed-loop phonocardiogram stimulation enables individuals to manage acute stress more effectively and whether the effects of this treatment persist for up to 4 weeks. The design of a further, longer-term, and more comprehensive study will help to substantiate claims regarding long-term cardiovascular or psychological outcomes.

This study did not include an acoustically equivalent sham arm (scrambled sound), thus limiting the ability to completely isolate the specific effects of real-time heart sounds from the non-specific effects of listening and directed attention. However, the comparison between the silent, metronome, and heart-sound arms provided a partial separation of these effects. The main objective of this study is to explore the overall efficacy of the intervention under real-world conditions rather than to isolate its precise mechanism of action. Future studies incorporating an acoustically matched sham arm are necessary to confirm the specificity of the proposed mechanism.

Another limitation is that the first session, the subsequent 10 sessions, and the day of the follow-up evaluation for all groups may not be conducted in the same season. As seasons are known to affect physiological parameters (Goswami et al., 2021; Goswami et al., 2020; Trozic et al., 2020), we will endeavor to conduct most recordings during a single season. This should prevent differences in ECG signals owing to seasonal changes.

Because of the front-end morphology of each participant, during measurements with fNIRS, not all 16 channels can produce significant signals. We will, therefore, use the maximum number of active channels for all participants. We will use a headband or cap to improve contact with the prefrontal area for all participants. At worst, 12 channels will provide sufficient information about the right, central, and left prefrontal cortex activity.

## 6 Conclusion

When subjected to a new stress test, traumatized people may show more pronounced changes in behavior, intolerance, and fatigue responses than before the trauma. This can lead to irreversible changes in the autonomic nervous system and the heart. This study aims to examine how participants cope with such situations and what type of biofeedback could be developed to manage them. In this context, victims’ emotional and psychological states will be monitored using a protocol based on biomedical (neurocardiac) signals and the sound of the person’s heart. This sound will be applied at a fixed frequency or in real time with allostatic auditory stimulation. A resonance that stabilizes the autonomic nervous system will thus provide biofeedback. ALLOSTAT0001 is an innovative project aimed at studying the medium- to long-term risks of trauma-induced stress in young participants. In this context, the emotional and psychological state of victims will be monitored using the protocol described, based on biomedical (neurocardiac) signals and the sound of the person’s heart. This sound will be applied at a fixed frequency or in real time with auditory stimulation. A resonance that stabilizes the autonomic nervous system will thus provide biofeedback. The ALLOSTAT0001 project aims to fill these gaps in this area of research.
